# Clinical characteristics of hospitalized male adolescents and young adults with avoidant/restrictive food intake disorder (ARFID)

**DOI:** 10.1186/s40337-024-01171-0

**Published:** 2025-01-08

**Authors:** Jason M. Nagata, Anita V. Chaphekar, Patrick Low, Ruben Vargas, Kyle T. Ganson, Anthony Nguyen, Sara M. Buckelew, Andrea K. Garber, Amanda E. Downey

**Affiliations:** 1 Department of Pediatrics, University of California, San Francisco, San Francisco, CA USA; 2 Department of Pediatrics, University of Cincinnati College of Medicine, Cincinnati, OH USA; 3 Factor-Inwentash Faculty of Social Work, University of Toronto, Toronto, ON Canada; 4 Department of Psychiatry and Behavioral Sciences, University of California, San Francisco, San Francisco, CA USA; 5 550 16th Street, 4th Floor, Box 0503, San Francisco, CA 94143 USA

**Keywords:** ARFID, Anorexia nervosa, Feeding and eating disorders, Female, Male, Refeeding

## Abstract

**Background:**

Avoidant/restrictive food intake disorder (ARFID) may result in significant medical sequelae. Compared to youth with eating disorders like anorexia nervosa (AN), youth with ARFID tend to be younger and are more likely to be male. We aim to describe sex differences in clinical characteristics of youth hospitalized for medical complications of ARFID and compare their characteristics with youth hospitalized for anorexia nervosa.

**Methods:**

This is a retrospective review of electronic medical records for youth with ARFID (*N* = 36; 13 male and 23 female) and AN (*N* = 355; 40 male and 315 female), including restricting and binge-eating/purging subtypes, aged 9–25 admitted to the inpatient UCSF Eating Disorders Program (2012–2020).

**Results:**

A greater proportion of youth with ARFID were male compared to youth with AN (36.1% vs. 11.2%). Male youth with ARFID (mean age 15.5 ± 2.8) had lower heart rate nadir (49.2 vs. 57.6 beats per minute, *p* = .019) and lower total cholesterol (129.8 vs. 159.3 mg/dL, *p* = .008), but higher hemoglobin (13.9 vs. 13.0 g/dL, *p* = .015) and prescribed calories at discharge (3323 vs. 2817 kcal, *p* = .001) compared to females with ARFID. Males with AN, who on average had higher admission BMI than males with ARFID (17.3 vs. 15.5 kg/m^2^, *p* = .013), required more (3785) kcal on discharge to restore medical stability than males with ARFID (3323 kcal). Compared to all youth with AN, youth with ARFID had lower body mass index (BMI, 15.7 vs. 17.0 kg/m^2^, *p* = .001) and lower vitamin D (26.5 vs. 33.0 ng/mL, *p* = .003).

**Conclusions:**

ARFID in males is associated with lower heart rate nadirs than in females with ARFID. Clinicians should be aware of unique medical complications in youth with ARFID compared to youth with AN.

## Abstract (plain language summary)

Adolescent and young adult males with avoidant/restrictive food intake disorder (ARFID) experience unique and significant medical complications compared to females with ARFID and youth with anorexia nervosa (AN). In this study, a greater proportion of youth with ARFID were male (36.1%) compared to those with AN (11.2%). Males with ARFID had a lower heart rate, lower total cholesterol, and higher hemoglobin levels than females with ARFID. They also required more nutrition at discharge to restore medical stability. Additionally, youth with ARFID of all sexes had lower BMI compared to youth with AN. These findings highlight important medical differences, particularly in males with ARFID, that clinicians should consider to guide appropriate treatment interventions in youth with ARFID.

## Introduction

 Avoidant/restrictive food intake disorder (ARFID) is an eating disorder included in the fifth edition of the Diagnostic and Statistical Manual of Mental Disorders (DSM-5) in 2013 [1]. Characterized by a persistent failure to meet appropriate nutritional needs unrelated to weight or shape concerns, ARFID can result in significant weight loss, nutritional deficiency, and interference with psychosocial functioning [2, 3]. Youth with ARFID tend to be of younger age and male sex compared to patients with other eating disorders such as anorexia nervosa (AN) [4, 5]. While more data are needed to understand the scope and epidemiology of ARFID, evidence suggests that more than half of youth with ARFID may require hospitalization for medical compromise related to the illness [6].

The medical complications of youth with ARFID requiring hospitalization have not been well characterized. Research examining adults with ARFID has identified malnutrition and electrolyte derangements as complications requiring prompt medical attention [7]. Leach et al. documented several complications in hospitalized adults with ARFID, including hypoglycemia, elevated liver function tests, hypophosphatemia, and electrocardiogram abnormalities [8]. Other laboratory abnormalities described in adults with ARFID include low vitamin D and leukopenia [7, 9].While limited data exist, youth with ARFID tend to require longer hospitalizations than youth with AN despite similar caloric intake [10]. A recent systematic review and meta-analysis of physical health complications in children and young people with ARFID reveals more normal heart rate and blood pressure compared to youth with AN, though it included both inpatient and outpatient samples [11]. Given these unique features, better characterization of the medical complications in hospitalized youth with ARFID will help to shape tailored treatment approaches.

Males with eating disorders are generally understudied compared to females, despite the increasing prevalence of eating disorders in all genders [12]. Males with eating disorders often present for medical care with significant laboratory and vital sign abnormalities requiring medical stabilization and hospital admission [13]. Furthermore, males require longer medical hospitalizations compared to females, likely due to higher metabolic requirements and the need for more nutrition to establish medical stability [14]. Anemia is observed in males with eating disorders more commonly than in females [14]. These sex differences, among others, highlight the need for sex-specific research addressing medical complications in patients with ARFID. To our knowledge, no investigation has assessed the medical complications of ARFID in hospitalized patients based on sex assigned at birth. This study aims to assess the medical complications and clinical characteristics in youth of both sexes hospitalized for medical complications from ARFID. To further characterize and contextualize youth with ARFID, we compare these sex differences to those seen in youth with AN.

## Methods

### Study population

We conducted a chart review of 601 youth, aged 9–25, admitted to the inpatient Eating Disorders Program at the University of California, San Francisco (UCSF) between May 2012 and August 2020. Youth diagnosed under DSM-IV criteria (8%) were reclassified to DSM-5 by our study team. All participants were hospitalized for medical complications related to ARFID (*n* = 36) and AN, including restricting and binge-eating/purging subtypes (*n* = 355). Those who did not meet DSM-5 criteria for ARFID or AN were excluded. The UCSF Inpatient Eating Disorders Program provides medical stabilization and diagnostic assessments by a tertiary, multidisciplinary team expert in eating disorder care. Indications for medical hospitalization were based on the Position Papers of the Society for Adolescent Health and Medicine (e.g., medical complications of malnutrition such as vital sign instability) [15, 16]. Daily caloric prescription is divided between three meals and three snacks, given on a schedule. The dietary macronutrient distribution conforms to the Dietary Guidelines for Americans 2020 (approximately 30–40% fat, 15–25% protein, and 35–55% carbohydrate) [17]. Patients have a set amount of time to complete their meal and snack in the presence of a ‘Patient Care Attendant’ who ensures meal completion. If unable to finish their meal or snack, patients must make up the calories they have missed with a nutritional replacement by mouth. If they do not complete the nutritional replacement, a nasogastric (NG) tube is placed.

### Study design

Data from the electronic medical record informed the demographic and physiologic characteristics of study participants. This data spanned demographics, illness duration, anthropometric measures, vital signs, and laboratory analyses from standard clinical documentation.

### Measurements

All measurements and evaluations followed established hospital protocols. At admission and throughout the hospitalization, vital signs were obtained. Lowest vital signs (e.g., heart rate, blood pressure) during the hospitalization were recorded as nadirs. Serum laboratory assessments were obtained at admission and each morning for the first seven days of the hospitalization. Analyses were conducted at a Clinical Laboratory Improvement Amendments (CLIA)-certified laboratory and adhered to the UCSF clinical laboratory’s reference ranges. Height measurements were performed using a wall-mounted stadiometer while patients were weighed in a hospital gown without undergarments on a standing scale before morning intake and post-voiding. Body mass index (BMI), presented as kg/m², was derived from initial weight and height measurements taken at admission. The percent median BMI (%mBMI) was calculated from the median BMI for age and sex using the Center for Disease Control growth charts [18]. Weight suppression at admission was assessed by subtracting the admission weight from the patient’s highest recorded weight and then dividing by the highest recorded weight. Duration of illness, length of hospital stay, and prescribed calories at admission and discharge were extracted by chart review. Licensed psychologists or psychiatrists made AN and ARFID diagnoses based on clinical assessments during the hospital admission. Refeeding hypophosphatemia, hypokalemia, or hypomagnesemia was defined as individuals with low phosphorous (< 3.0 mg/dL), potassium (< 3.5 mmol/L), or magnesium (< 1.8 mg/dL), respectively, after 24 h of hospitalization (hospital day 2 through day of discharge) and the initiation of refeeding based on a published multicenter refeeding protocol [19].

### Ethics

This retrospective chart review study involving human participants was in accordance with the ethical standards of the institutional and national research committee and with the 1964 Helsinki Declaration and its later amendments or comparable ethical standards. The Institutional Review Board of the UCSF approved this study (20-30323).

### Statistical analysis

Using Stata 18.0 (StataCorp LP, College Station, TX), we report descriptive statistics of the demographic and clinical factors for youth with ARFID and AN, stratified by sex. We then compared groups using independent samples t-tests (continuous variables) and chi-squared or Fisher’s exact tests (categorical variables). Group comparisons were for males with ARFID vs. females with ARFID, males with ARFID vs. males with AN, and females with ARFID vs. females with AN. Given multiple comparisons, we implemented the Benjamini-Hochberg procedure to adjust for a false discovery rate [20]. We conducted linear regression analyses with diagnosis (ARFID vs. AN) or sex (male vs. female) as the exposure variable and heart rate nadir as the outcome variable, adjusting for BMI.

## Results

Table 1 presents the demographic and clinical characteristics of hospitalized adolescents and young adults with ARFID and AN by sex. Among males with ARFID (*n* = 13), the average (SD) age was 15.5 (2.8) years, BMI was 15.5 (1.6) kg/m^2^, and %mBMI was 76.7% (10.3%). The average prescribed daily calorie intake was 1923 (277) kcal at admission and increased to 3323 (396) kcal at discharge. The average duration of illness prior to hospitalization was 14.6 (15.1) months, and the average length of hospital stay was 9.5 (3.2) days. The most common serum laboratory abnormalities in males with ARFID on admission included vitamin D insufficiency (33.3%) or deficiency (16.7%), anemia (30.8%), hypokalemia (15.4%), zinc deficiency (8.3%), and elevated alanine transaminase (ALT, 7.7%).

Males with ARFID had lower heart rate nadir (49.2 vs. 57.6 beats per minute, *p* = .019), lower total cholesterol (129.8 vs. 159.3 mg/dL, *p* = .008), and lower triglycerides (46.8 vs. 80.1 mg/dL, *p* < .001), but higher hemoglobin (13.9 vs. 13.0 g/dL, *p* = .015) and prescribed calories at discharge (3323 vs. 2817 kcal, *p* = .001) compared to females with ARFID.

ARFID subtype is presented in Table 2. The most common presentation was concern about aversive consequences of eating (55.6% overall), followed by sensory (25.0% overall), and lack of interest in eating or food (11.1%). There were no significant sex differences in the presentation subtype.

**Table 1:** Demographic and clinical characteristics of adolescents and young adults hospitalized with avoidant/restrictive food intake disorder (ARFID) and anorexia nervosa by sex

Characteristic	ARFID					Anorexia Nervosa									
	Total (N = 36)	Male (n = 13)	Female (n = 23)	p ^a^	Effect size^b^	Total (N = 355)	p ^c^	Effect size^b^	Male (n = 40)	p ^d^	Effect size^b^	Female (n = 315)	p ^e^	Effect size^b^	
Age, years, mean (SD)	15.3 ± 3.0	15.5 ± 2.8	15.2 ± 3.1	.760	0.107	15.7 ± 2.8	.474	0.125	16.2 ± 2.7	.468	0.234	15.6 ± 2.8	.507	0.143	
Race/ethnicity, n (%)				.923	0.016		.266	0.056		.691	0.055		.251	0.063	
Asian or NHOPI^f^	1 (2.8)	0 (0)	1 (4.4)			32 (9.0)			4 (10.0)			28 (8.9)			
Hispanic	9 (25.0)	2 (15.4)	7 (30.4)			58 (16.3)			12 (30.0)			46 (14.6)			
Multiracial	3 (8.3)	1 (7.7)	2 (8.7)			11 (3.1)			1 (2.5)			10 (3.2)			
Non-Hispanic Black or African American	3 (8.3)	2 (15.4)	1 (4.4)			5 (1.4)			1 (2.5)			4 (1.3)			
Non-Hispanic White	19 (52.8)	7 (53.9)	12 (52.2)			221 (62.3)			19 (47.5)			202 (64.1)			
Other	0 (0)	0 (0)	0 (0)			17 (4.8)			2 (5.0)			15 (4.8)			
Unknown/Declined	1 (2.8)	1 (7.7)	0 (0)			11 (3.1)			1 (2.5)			10 (3.2)			
BMI, kg/m^2^, mean (SD)	15.7 ± 1.8	15.5 ± 1.6	15.8 ± 2.0	.764	0.133	17.0 ± 2.3	**.001**	0.564	17.3 ± 2.3	**.013**	0.824	16.9 ± 2.3	**.021**	0.502	
Percent median BMI, mean (SD)	78.2 ± 9.8	76.7 ± 10.3	79.0 ± 9.6	.502	0.235	83.8 ± 10.9	**.003**	0.522	83.5 ± 11.6	.065	0.602	83.8 ± 10.8	**.038**	0.450	
Percent weight suppression at admission, mean (SD)^g^	12.7 ± 9.9	9.0 ± 5.7	14.6 ± 11.1	.151	0.572	20.1 ± 10.4	**< .001**	0.714	22.0 ± 11.4	**.001**	1.237	19.9 ± 10.2	**.027**	0.514	
Duration of illness in months, mean (SD)^g^	16.3 ± 15.1	14.6 ± 15.1	17.0 ± 15.4	.684	0.163	15.3 ± 18.7	.761	0.057	14.9 ± 13.6	.953	0.022	15.3 ± 19.2	.682	0.091	
Lowest vital signs during hospitalization															
Heart rate (beats per minute)	54.6 ± 10.5	49.2 ± 9.6	57.6 ± 9.9	**.019**	0.855	44.5 ± 10.2	**< .001**	0.991	42.3 ± 10.5	**.041**	0.671	44.7 ± 10.1	**< .001**	1.273	
Heart rate < 50 beats per minute	9 (25.0)	5 (38.5)	4 (17.4)	.161	0.234	273 (69.8)	**< .001**	0.311	38 (71.7)	**.002**	0.421	231 (73.3)	**< .001**	0.306	
Systolic blood pressure (mmHg)	85.7 ± 8.5	86.9 ± 9.1	85.0 ± 8.3	.513	0.229	82.2 ± 9.3	**.033**	0.374	82.4 ± 12.1	.222	0.395	82.2 ± 8.9	.150	0.312	
Systolic blood pressure < 90 mmHg	26 (72.2)	9 (69.2)	17 (73.9)	.763	0.050	279 (78.6)	.379	0.045	26 (65.0)	.780	0.038	253 (80.3)	.460	0.040	
Diastolic blood pressure (mmHg)	46.9 ± 5.6	47.6 ± 5.1	46.4 ± 6.0	.554	0.207	44.8 ± 6.8	.074	0.313	43.4 ± 8.2	.086	0.559	44.9 ± 6.6	.292	0.228	
Diastolic blood pressure < 45 mmHg	11 (30.6)	3 (23.1)	8 (34.8)	.464	0.122	154 (43.4)	.138	0.075	19 (47.5)	.121	0.213	135 (42.9)	.449	0.041	
Admission serum electrolyte laboratory values, mean (SD)															
Magnesium (mg/dL)	2.1 ± 0.2	2.0 ± 0.2	2.2 ± 0.2	.159	0.500	2.1 ± 0.2	.375	0.155	2.1 ± 0.2	.100	0.535	2.1 ± 0.2	.756	0.067	
Hypomagnesemia (< 1.8 mg/dL)	1 (2.8)	1 (7.7)	0 (0)	.177	0.225	5 (1.4)	.524	0.032	0 (0)	.077	0.243	5 (1.6)	.543	0.033	
Phosphorus (mg/dL)	3.9 ± 0.6	4.0 ± 0.8	3.9 ± 0.5	.670	0.149	3.9 ± 0.6	.660	0.077	3.9 ± 0.7	.822	0.072	3.9 ± 0.6	.871	0.035	
Hypophosphatemia (< 3.0 mg/dL)	2 (5.6)	1 (7.7)	1 (4.4)	.674	0.070	20 (5.6)	.985	0.001	3 (7.5)	.982	0.003	17 (5.4)	.829	0.012	
Potassium (mmol/L)	3.8 ± 0.4	3.9 ± 0.5	3.7 ± 0.3	.173	0.483	3.9 ± 0.5	.442	0.135	3.9 ± 0.5	.682	0.132	3.9 ± 0.5	.189	0.284	
Hypokalemia (< 3.5 mmol/L)	6 (16.7)	2 (15.4)	4 (17.4)	.877	0.026	54 (15.2)	.817	0.012	4 (10.0)	.595	0.073	50 (15.9)	.848	0.010	
Refeeding electrolyte laboratory values, n (%)															
Refeeding hypomagnesemia (< 1.8 mg/dL)	4 (11.1)	1 (7.7)	3 (13.0)	.624	0.082	74 (20.9)	.164	0.070	7 (17.5)	.391	0.118	67 (21.3)	.347	0.051	
Refeeding hypophosphatemia (< 3.0 mg/dL)	2 (5.7)	0 (0)	2 (8.7)	.274	0.182	31 (8.7)	.514	0.033	2 (5.0)	.411	0.113	29 (9.2)	.935	0.005	
Refeeding hypokalemia (< 3.5 mmol/L)	5 (13.9)	2 (15.4)	3 (13.0)	.845	0.033	42 (11.8)	.718	0.018	4 (10.0)	.595	0.073	38 (12.1)	.889	0.008	
Combined refeeding electrolyte deficiencies^h^	9 (25.0)	3 (23.1)	6 (26.1)	.841	0.033	100 (28.2)	.686	0.020	10 (25.0)	.889	0.019	90 (28.6)	.799	0.014	
Other laboratory evaluation at admission (normal range)															
Total cholesterol, mg/dL^g^	146.5 ± 31.3	129.8 ± 25.0	159.3 ± 30.1	**.008**	1.054	170.7 ± 44.5	**.004**	0.554	161.3 ± 47.2	**.026**	0.737	171.8 ± 44.1	.248	0.289	
High total cholesterol (≥ 200 mg/dL)	1 (3.3)	0 (0)	1 (5.9)	.374	0.162	66 (19.6)	**.027**	0.116	6 (15.8)	.127	0.214	60 (20.1)	.148	0.082	
Triglycerides, mg/dL^g^	65.6 ± 27.1	46.8 ± 6.8	80.1 ± 28.1	**< .001**	1.536	71.3 ± 36.8	.412	0.157	71.9 ± 34.9	**.014**	0.822	71.2 ± 37.0	.334	0.242	
High triglycerides (≥ 130 mg/dL)	1 (3.3)	0 (0)	1 (5.9)	.374	0.162	24 (7.3)	.418	0.043	4 (10.5)	.223	0.171	20 (6.8)	.880	0.009	
Hemoglobin, g/dL^g^	13.3 ± 1.2	13.9 ± 1.1	13.0 ± 1.1	**.015**	0.894	12.8 ± 1.2	**.040**	0.366	13.4 ± 1.2	.244	0.379	12.8 ± 1.1	.597	0.117	
Hematocrit, g/dL^g^	38.8 ± 2.9	39.9 ± 2.8	38.1 ± 2.7	.073	0.648	37.9 ± 3.3	.104	0.289	39.0 ± 3.5	.415	0.264	37.7 ± 3.2	.558	0.130	
Anemic (M: < 13.6 g/dL; F: < 11.8 g/dL)	7 (20.0)	4 (30.8)	3 (13.6)	.221	0.207	81 (23.6)	.636	0.024	22 (57.9)	.091	0.237	59 (19.3)	.514	0.036	
Zinc (plasma), mcg/dL^g^	65.3 ± 10.2	64.8 ± 10.5	65.6 ± 10.2	.850	0.070	64.2 ± 15.1	.679	0.077	66.8 ± 18.3	.732	0.115	63.8 ± 14.7	.602	0.121	
Low Zinc (plasma) (< 55 mcg/dL)	3 (9.4)	1 (8.3)	2 (10.0)	.876	0.028	76 (23.7)	.064	0.099	9 (24.3)	.232	0.171	67 (23.6)	.161	0.080	
25-hydroxyvitamin D, ng/mL^g^	26.5 ± 8.9	29.7 ± 8.8	24.6 ± 8.5	.115	0.593	33.0 ± 12.0	**.003**	0.557	31.4 ± 11.5	.629	0.160	33.3 ± 12.1	**.002**	0.731	
25-hydroxyvitamin D Insufficiency (20–29 ng/mL)	13 (40.6)	4 (33.3)	9 (45.0)	.515	0.115	101 (30.2)	.226	0.063	6 (15.0)	.158	0.196	95 (32.3)	.243	0.066	
Deficiency (< 20 ng/mL)	7 (21.9)	2 (16.7)	5 (25.0)	.581	0.098	35 (10.5)	.053	0.101	8 (20.0)	.797	0.036	27 (9.2)	**.024**	0.128	
Severe deficiency (< 12 ng/mL)	2 (6.3)	0 (0)	2 (10.0)	.258	0.200	9 (2.7)	.260	0.059	4 (10.0)	.254	0.158	5 (1.7)	**.015**	0.137	
Creatinine (0.45–1.08 mg/dL)	0.6 ± 0.1	0.7 ± 0.2	0.6 ± 0.1	.079	0.629	0.7 ± 0.2	**< .001**	0.594	0.8 ± 0.2	**.027**	0.725	0.7 ± 0.1	**< .001**	0.772	
Blood urea nitrogen (7–21 mg/dL)^g^	11.1 ± 4.1	12.0 ± 4.4	10.7 ± 4.0	.377	0.319	13.5 ± 6.0	**.027**	0.394	17.3 ± 11.7	.132	0.505	13.0 ± 4.7	**.024**	0.492	
Thyroid stimulating hormone (0.45–4.33 mIU/L)^g^	2.0 ± 1.6	1.7 ± 0.8	2.2 ± 1.9	.301	0.367	2.0 ± 1.4	.926	0.016	2.1 ± 1.3	.194	0.425	2.0 ± 1.4	.420	0.179	
Abnormal TSH (< 0.45 or > 4.33 mIU/L)	2 (5.7)	0 (0)	2 (9.1)	.263	0.189	21 (6.3)	.900	0.007	3 (8.1)	.290	0.150	18 (6.0)	.565	0.032	
Free thyroxine (10–18 pmol/L)^g^	13.5 ± 1.9	13.8 ± 1.9	13.3 ± 2.0	.467	0.258	11.6 ± 1.8	**< .001**	1.010	11.9 ± 1.9	**.004**	0.978	11.6 ± 1.8	**< .001**	0.932	
Abnormal free thyroxine (< 10 or > 18 pmol/L)	1 (2.9)	1 (7.7)	0 (0)	.187	0.223	23 (6.9)	.358	0.048	2 (5.6)	.783	0.039	21 (7.1)	.198	0.072	
Aspartate transaminase (AST), U/L^g^	23.5 ± 7.8	23.9 ± 4.9	23.2 ± 9.2	.791	0.094	30.3 ± 40.3	.316	0.178	53.2 ± 107.8	.336	0.310	27.3 ± 15.6	.228	0.267	
Elevated aspartate transaminase (> 35 U/L)	2 (5.7)	0 (0)	2 (9.1)	.263	0.189	43 (12.7)	.229	0.062	11 (27.5)	**.034**	0.292	32 (10.7)	.816	0.013	
Alanine transaminase (ALT), U/L^g^	17.3 ± 13.8	16.1 ± 5.0	18.1 ± 17.1	.683	0.144	23.8 ± 33.1	.256	0.202	44.0 ± 80.6	.221	0.396	21.1 ± 18.1	.458	0.164	
Elevated alanine transaminase (> 24 U/L)	5 (14.3)	1 (7.7)	4 (18.2)	.392	0.145	81 (23.9)	.198	0.067	16 (40.0)	**.030**	0.298	65 (21.7)	.695	0.022	
Albumin (3.5–5.0 g/dL)^g^	4.2 ± 0.4	4.3 ± 0.4	4.2 ± 0.5	.419	0.286	4.3 ± 0.4	.210	0.223	4.3 ± 0.4	.985	0.006	4.3 ± 0.4	.125	0.340	
Clinical and nutritional characteristics															
Length of stay in days, mean (SD)	9.5 ± 5.7	9.5 ± 3.2	9.5 ± 6.7	.993	0.003	10.3 ± 6.7	.479	0.124	11.2 ± 5.7	.339	0.308	10.2 ± 6.8	.623	0.106	
Prescribed kcal, mean (SD)															
Admission	1944 ± 256	1923 ± 277	1957 ± 248	.712	0.129	1972 ± 222	.479	0.124	2010 ± 235	.273	0.354	1968 ± 220	.817	0.050	
Discharge	3000 ± 448	3323 ± 396	2817 ± 371	**.001**	1.330	3139 ± 533	.132	0.264	3785 ± 720	**.032**	0.702	3057 ± 443	**.012**	0.546	

**Bold** indicates statistical significance after implementing the Benjamini-Hochberg procedure

P-value is for t-tests for continuous variables and Pearson’s chi square tests (or Fisher’s exact test as appropriate) for categorical variables. For race/ethnicity, p-value is for Pearson’s chi square test comparing a binary race/ethnicity variable (non-Hispanic white vs. all other race/ethnicities)

^a^ P for comparison of males with ARFID compared to females with ARFID

^b^ Cohen’s d for continuous variables, Cramer’s V for categorical variables

^c^ P for comparison of patient sample with ARFID compared to patient sample with anorexia nervosa

^d^ P for comparison of males with ARFID compared to males with anorexia nervosa

^e^ P for comparison of females with ARFID compared to females with anorexia nervosa

^f^ NHOPI = Native Hawaiian and Other Pacific Islanders

^g^ Some sample sizes (n) for the clinical data do not sum up to the total population (N) due to missing data

^h^ A variable indicating refeeding hypomagnesemia or hypophosphatemia or hypokalemia

**Table 2:** Avoidant/restrictive food intake disorder (ARFID) presentation subtype by sex

ARFID subtype, *n* (%)	Total (*N* = 36)	Male (*n* = 13)	Female (*n* = 23)	*p* ^a^	Effect size^b^
				0.650	0.237
Concern about aversive consequences of eating	20 (55.6)	6 (46.2)	14 (60.9)		
Sensory	9 (25.0)	4 (30.8)	5 (21.7)		
Lack of interest in eating or food	4 (11.1)	1 (7.7)	3 (13.0)		
Unknown	3 (8.3)	2 (15.4)	1 (4.4)		

^a^ P for comparison of males with ARFID compared to females with ARFID. P-value is for Fisher’s exact test

^b^ Cramer’s V for categorical variables

Overall, as compared to youth with AN, youth with ARFID had lower body mass index (BMI, 15.7 vs. 17.0 kg/m^2^, *p* = .001), percent median BMI (78.2 vs. 83.8 kg/m^2^, *p* = .003), weight suppression (12.7% vs. 20.1%, *p* < .001), total cholesterol (146.5 vs. 170.7 mg/dL, *p* = .004), and vitamin D (26.5 vs. 33.0 ng/mL, *p* = .003), but higher heart rate nadir (54.6 vs. 44.5 beats per minute, *p* < .001), systolic blood pressure nadir (85.7 vs. 82.2 mmHg, *p* = .033), hemoglobin (13.3 vs. 12.8 g/dL, *p* = .040), and free thyroxine (13.5 vs. 11.6 pmol/L, *p* < .001). Interestingly, males with AN, who on average had higher admission BMI than males with ARFID (17.3 vs. 15.5 kg/m^2^, *p* = .013), required more (3785) kcal on discharge to restore medical stability than males with ARFID (3323 kcal). Compared to males with AN, males with ARFID had less weight suppression on admission (9.0% vs. 22.0%, *p* = .001).

We conducted several regression analyses with heart rate nadir as the outcome, adjusting for BMI. Among youth with ARFID, male sex was associated with an 8.80 (95% CI 2.14, 15.46) lower heart rate nadir after adjusting for BMI. Among male youth, anorexia nervosa diagnosis (compared to ARFID) was associated with a 5.38 lower (95% CI -1.62, 12.37) heart rate nadir but the difference was not statistically significant. Among female youth, anorexia nervosa diagnosis (compared to ARFID) was associated with a 13.02 lower (95% CI 8.69, 17.36) lower heart rate nadir. Among the total sample, anorexia nervosa diagnosis (compared to ARFID) was associated with a 10.12 lower (95% CI 6.56, 13.69) heart rate nadir.

## Discussion

The current study offers a novel examination of the clinical characteristics and medical complications associated with ARFID in hospitalized youth by sex. Notably, males with ARFID experienced unique medical complications relative to females, including lower heart rate nadir, total cholesterol, and triglycerides, but higher hemoglobin and prescribed calories at discharge. Relative to patients with AN, patients with ARFID had lower BMI, percent median BMI, total cholesterol, and vitamin D, but higher heart rate nadir, systolic blood pressure nadir, hemoglobin, and free thyroxine. These findings underscore the unique medical sequela found in hospitalized adolescents and young adults with ARFID, and the need to tailor medical treatment that best addresses their medical compromise.

Males with ARFID had lower heart rate nadirs compared to females with ARFID, which has similarly been observed between male and female youth with atypical AN [21]. Bradycardia is a known complication of the malnutrition associated with ARFID and is postulated to be a physiologic adaptation to increased vagal tone and decreased metabolism from low caloric energy intake, similar to other restrictive eating disorders [8, 22]. Our finding of lower heart rate nadirs in males compared to females with ARFID may reflect higher metabolic needs in males and more severe malnutrition from insufficient caloric intake [23]. In addition, males with AN had lower heart rate nadirs compared to males with ARFID. Future studies may explore the duration of malnutrition as contributing to the degree of bradycardia by sex. Whether bradycardia from malnutrition increases the risk of prolongation of the QTc interval and other life-threatening arrhythmias is a topic of ongoing debate [24, 25]. Clinicians can consider cardiac monitoring in hospitalized male patients with ARFID, particularly during refeeding and recovery phases, to mitigate potential complications.

Our data were consistent with prior work characterizing hospitalized youth with ARFID. Our sample presented with less weight suppression, though lower absolute BMI, compared to youth with AN, as in another study [10]. ARFID develops as an eating disturbance due to sensory sensitivities, fear of adverse consequences of eating (e.g. vomiting or choking), and/or a general lack of interest in food. We found that fear of adverse consequences of eating was the most common subtype in our sample, followed by sensory concerns and then lack of interest in food for both males and females. This mechanism yielding malnutrition tends to be a long-standing developmental concern, distinct from the body image-related motivations seen in AN, where patients actively seek to reduce body weight [26]. Thus, decreased weight suppression may reflect a more chronic course of malnutrition. Indeed, the smaller deviation from premorbid growth trajectory can lead to delays in detection and a protracted course of illness [27]. Further, we found that males with ARFID, despite having a lower absolute BMI on admission compared to males with AN, required fewer calories to restore medical stability. Dividing the average calories required to restore medical stability by the average body mass index between males with ARFID and males with AN, nearly the same caloric load per unit of BMI was required to restore medical stability. Thus, the overall difference in caloric requirement is likely a function of differences in total body mass, metabolic rate, or other physiological factors influencing energy expenditure and refeeding needs between the two groups.

Together with serum laboratory and vital sign differences suggesting unique medical sequelae, our data further support the need to tailor medical management for youth with ARFID [28]. Prior studies conclude that a standard eating disorder protocol applied broadly across eating disorder diagnoses yields longer lengths of stay and more nasogastric tube insertions for youth with ARFID [10, 28]. To optimize medical and psychological recovery for youth with ARFID, our data further support tailored inpatient medical management distinct from those protocols developed for AN. For example, because youth with ARFID have less acute weight suppression as compared to their AN counterparts, more aggressive refeeding approaches may be less likely to precipitate refeeding syndrome. Similarly, males with ARFID may start at higher kilocalories on admission compared to females, recognizing their sex-specific metabolic demands are higher than for females. Future studies are needed to understand the implications of tailored refeeding approaches.

Our investigation has several limitations. First, the sample size of male patients with ARFID is small which may limit the generalizability of findings and fail to capture variability within this population. Future studies would benefit from larger, more balanced samples for robust analyses, including consideration of gender differences beyond sex assigned at birth. Second, this retrospective study design limits any causal inferences. Additionally, our study was conducted from a single tertiary care hospital in Northern California, and our findings may not be generalizable to other populations. Finally, we do not have data on whether patients required NG tube feedings, which may significantly differ between those with AN and ARFID and may change the length of hospitalization. This is an important area of future research. Additional future investigations should consider larger study populations and a prospective study design to further elucidate the relationship between sex and medical complications of ARFID.

## Conclusion

We found sex differences in the clinical characteristics in youth hospitalized for medical complications of ARFID, as well as clinical characteristics that differ from youth with AN. Clinicians should be aware of these sex-specific findings in the diagnosis and management of ARFID. Eating disorders in males remain an under-researched public health concern. Future research should validate our findings using broader samples and investigate targeted intervention strategies in this population.

## Data Availability

The data that support the findings of this study are available on request from the corresponding author, J.M.N. The data are not publicly available due to confidentiality restrictions (e.g., their containing information that could compromise the privacy of research participants).

## Abbreviations

ARFID: Avoidant/restrictive food intake disorder
CLIA: Clinical Laboratory Improvement Amendments
DSM-5: Diagnostic and Statistical Manual of Mental Disorders
BMI: Body mass index
